# Combinatory Effects of Training and Nutritive Administration of Carbohydrates and Protein via Food on Strength in Postmenopausal Women, and Old Men and Women

**DOI:** 10.3390/nu15061531

**Published:** 2023-03-22

**Authors:** Katharina Hofmann, Ulrich Flenker, Gina Kiewardt, Patrick Rene Diel

**Affiliations:** Institute for Cardiovascular Research and Sports Medicine, Department of Molecular and Cellular Sports Medicine, German Sports University, Am Sportpark Müngersdorf 6, 50933 Cologne, Germany

**Keywords:** protein/carbohydrate supplementation, sling training, endurance training, strength training, BMI

## Abstract

The age-related loss of muscle mass promotes many impairments. Training and protein supplementation are suggested to prevent muscle wasting, but recommendations for all populations are not based on scientific evidence. This study combines protein/carbohydrate supplementation (PCS) and training for seniors and postmenopausal women. Project A: 51 postmenopausal women (PMW, 57.3 ± 3.0 years old) underwent health-oriented training (12 weeks, moderate-strength training + moderate-endurance training). The intervention group (IG) additionally received 110 g sour milk cheese (SMC) and toast. Project B: 25 women and 6 men (65.9 ± 4.9 years old) performed intense sling training (12 weeks). The IG additionally received 110 g SMC, toast, and buttermilk. Strength was tested before and after in both studies. Project A: there was significant increase in strength, no additional effect of PCS, and a reduction in body fat in the controls. Project B: there was significant increase in strength, significant additional effects of PCS for trunk strength, and a significant reduction in body weight. Combining training and PCS may counteract strength loss. Combined endurance/resistance training is recommended to PMW for whom the benefits of PCS are restricted. Aged subjects may benefit from PCS when training intensely, but these benefits may be strongly individual.

## 1. Introduction

Aging is accompanied by a variety of physical changes, such as a decrease in muscle mass up to sarcopenia, an increase in cardiovascular diseases, and frailty syndrome. In women, the onset of menopause or menopausal transition plays a role. Likewise, the massive decrease in estradiol supports the decrease in muscle mass and promotes the development of sarcopenia [1,2,3]. The likelihood of metabolic diseases such as Type 2 diabetes mellitus and metabolic syndrome also strongly increases [1,4]. There are numerous studies demonstrating the beneficial effects of exercise in the prevention and therapy of muscle mass loss, sarcopenia, metabolic syndrome, and risk for cardiovascular diseases [5,6,7,8,9,10,11].

The reduction in muscle mass during aging is due to a decrease in physical activity, and an imbalance between muscle-protein synthesis and breakdown. This can lead to the development of sarcopenia [10,12], which negatively impacts the functional capacity and quality of life of affected persons [12,13]. The revised 2019 version of the guidelines of the European Working Group on Sarcopenia in Older People (EWGSOP) emphasizes muscle strength as the main determinant, as it is best suited for predicting the adverse outcomes of sarcopenia [14]. Naseeb and Volpe summarized that protein supplementation and long-term aerobic exercise reduce the age-related loss of muscle strength [15]. An age-related decrease in muscle mass, even if it cannot be defined as sarcopenia, is a general risk in the aging population [15,16] that menopause promotes [16]. Avoiding a decrease in muscle mass and strength with physical training [17] is important for the prevention of a variety of age-related diseases. Aging is often accompanied by a decrease in physical activity [10]. In many cases, musculoskeletal disorders such as osteoarthritis cause this [17,18], but there are various additional reasons, including psychological ones [19,20]. Untreated, these impairments lead to an increased risk of becoming frail. Frailty syndrome is characterized by reduced activity and gait speed, a decrease in body strength, fatigue, and weight loss. Sarcopenia, stroke, myocardial infarction, arterial hypertension, and diabetes mellitus are closely related to the syndrome. A reduction in risk factors, and endurance, strength, and coordination training affect the risk of frailty syndrome [8]. The beneficial effects of physical activity on the maintenance of skeletal muscle mass are supported with protein supplementation [10]. There is a difference between protein supplementation that aims to compensate for the lack of protein intake through a normal diet and a situation with higher protein needs. The first scenario is typical for geriatric or cachectic individuals [21]. The adequate plasma levels of essential amino acids have a positive effect on muscle protein synthesis [22]. Chronic inflammatory processes that are exacerbated in old age by the decrease in estrogen and the increase in visceral adipose tissue favor proteolysis over protein synthesis. Thus, the breakdown of dietary proteins is imbalanced with the formation of new proteins from amino acids in cells. The result is an increased demand for proteins for equivalent and sufficient muscle protein synthesis in older adults [21,23]. A 2015 data analysis conducted by Gregorio et al. on postmenopausal women identified that about 25% of the population had lower protein intake than the daily recommendation [24]. This same subgroup showed a significant limitation in upper- and lower-extremity functionality. However, for the majority of postmenopausal women and older individuals, protein via supplementation is not needed as a strategy to compensate for a lack of protein uptake. Nevertheless, protein uptake in such individuals, as in younger ones, can support the functional adaptation of the skeletal muscle to a training stimulus [25]. After exercise, the intake of an additional 20–25 g of protein is recommended [21]. Amino acid availability shows a positive effect on muscle development, lean and muscle mass, and muscle strength. Likewise, it increases the plasma concentrations of IGF-1. Protein intake also positively influences calcium absorption and, thus, supports bone health [2]. Studies showed that the combined intake of proteins and carbohydrates leads to higher glycogen storage in the skeletal muscle, and a higher increase in blood sugar and insulin concentration than those of just carbohydrate combinations [26]. The increase in serum insulin levels entails the binding of insulin to IGF-1 receptors, which stimulates muscle protein synthesis in the skeletal muscle and the uptake of amino acids into skeletal muscle cells via various processes [27]. Isenmann et al. compared the intake of a protein/carbohydrate combination via shakes and natural foods by directly following a workout with regard to the regenerative effect on the muscles. The results showed that shakes and supplementation via a natural protein source could equally reduce muscle damage after exercise, and insulin was involved in the regenerative effects [27]. Lichtenberg and colleagues showed that training with protein supplementation using powders also resulted in significant muscle and strength gains in sarcopenic seniors [28]. In those studies, however, supplementation was always via powders or capsules and never via food. In addition, the studies in the available reviews were not consistent. Different supplements, compositions, and time points were used [29,30]. Trommelen et al. indicated that age, and the type and timing of supplementation play a decisive role, so supplementation should be specifically adapted to, for example, age [31]. Eating dairy products and white bread has proregenerative effects on skeletal muscle after exercise [32]. On the basis of the work of Diel and colleagues, and Isenmann and colleagues, this study examines whether the combined uptake of protein and carbohydrates directly after training from natural protein sources could also result in an increase in training adaption, mainly muscle strength, in postmenopausal women and old individuals.

## 2. Materials and Methods

### 2.1. Study A: Effect of Training and Protein/Carbohydrate Supplementation in Postmenopausal Women

#### 2.1.1. Study Design and Participants

**Figure 1:**
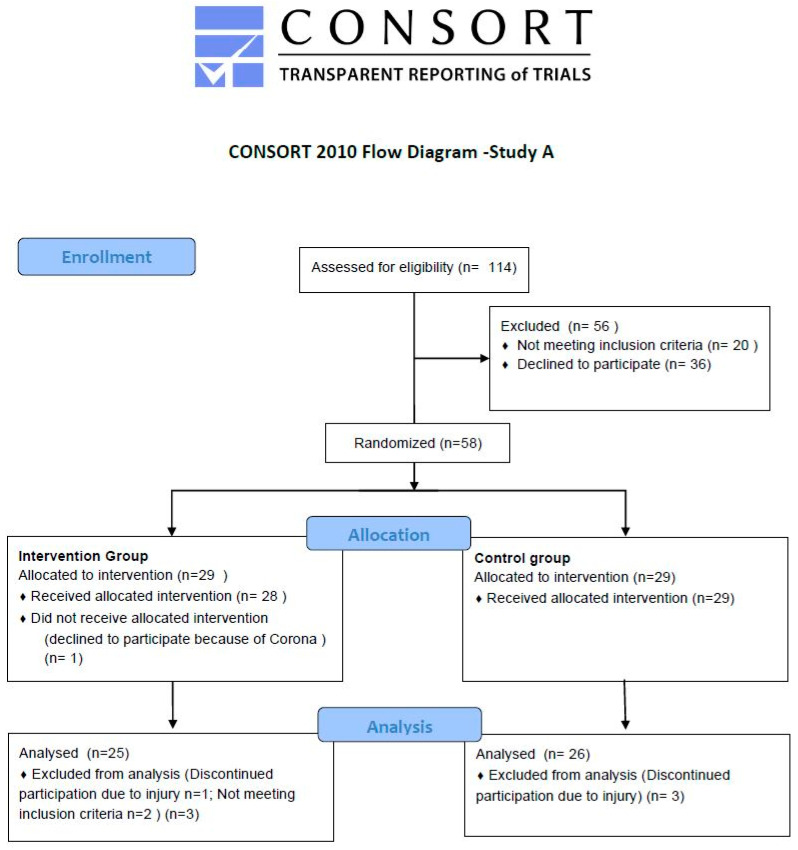
Consort flow diagram—Study A.

The study was a randomized intervention with 2 (CG and IG) parallel groups. We recruited 58 women between 50 and 65 years old in Germany by using personal contacts, calls on social media, gynecologists as gatekeepers, or the newsletter of the German Menopause Society (Figure 1). The sample size was determined on the basis of preliminary studies by Wacker [33]. The study started in January 2021 and was completed in November 2021. All examinations took place at the German Sport University, Cologne under the current valid COVID-19 protection regulations. Inclusion criteria were postmenopausal status, and the last menstrual period had to have been at least two years earlier. Exclusion criteria were hormonal diseases, metabolic diseases, cardiac arrhythmias requiring treatment, and limiting neurological, muscular, degenerative, or gastrointestinal diseases. Participants with a history of cancer within the past 5 years were excluded. Unbalanced diets such as vegan diets, smoking, and hormonal substitutions of any kind were excluded. All women had a low-to-moderate fitness status and none of them exercised more than twice per week in terms of strength or endurance training. Prior to recruitment, the approval of the ethics committee of the German Sport University, Cologne (number 008/2021) was obtained, and the study was registered in the German Clinical Trials Register, number DRKS-ID: DRKS00024144. The study protocol was in accordance with the Declaration of Helsinki. After the study procedure had been communicated via telephone, and the inclusion and exclusion criteria had been checked, the women received all information in paper form. Subsequently, an appointment was booked to sign the informed consent form, clarify questions, and start with the examinations. All 63 participants signed the informed consent form.

#### 2.1.2. Test Day

All participants fasted, with their last meal 12 h before the examination. After the blood samples had been collected, anthropometric data (weight, height, abdominal girth, and body composition) were collected via bioimpedance analysis (BIA) (BodyExplorer, Kommunikation & Service GmbH, Berliner Chaussee 74, 15234 Frankfurt, Oder). The blood was analyzed by the Wisplinghoff laboratory; parameters to determine postmenopausal status were estradiol, progesterone, and follicle stimulating hormone (FSH). To determine endurance capacity, a lactate threshold test was performed on a treadmill (Woodway PPS55med, Woodway GmbH, Steinackerstr. 20, 79576 Weil am Rhein): participants started at 5 km/h, and the speed was increased by 1.2 km/h every 5 min. Termination criteria for the test were reaching the maximal heart rate (220 minus age), feeling unwell or exhausted, or reaching 20 in the BORG scale. Hand-grip strength was tested via a grip test; to determine the maximal force for the chest via a chest press and leg strength via a leg press, the repetition maximum was tested according to Rühl [34]. According to Rühl, 1RM is calculated based on preformed repetitions.

All participants were randomly divided into an intervention group and a control group using a computer program (RITA version 1.51) while taking into account the parameters of age, weight, and walking speed (km/h) at 60% of the 4 mmol lactate/threshold. Before randomization, participants were stratified by age (<55, 55–60, >60 years old), by weight (<70, 70–90, >90 kg), and walking speed (km/h) at 60% of the 4 mmol/threshold (<4, 4–5, >5 km/h).

#### 2.1.3. Training Intervention

**Figure 2:**
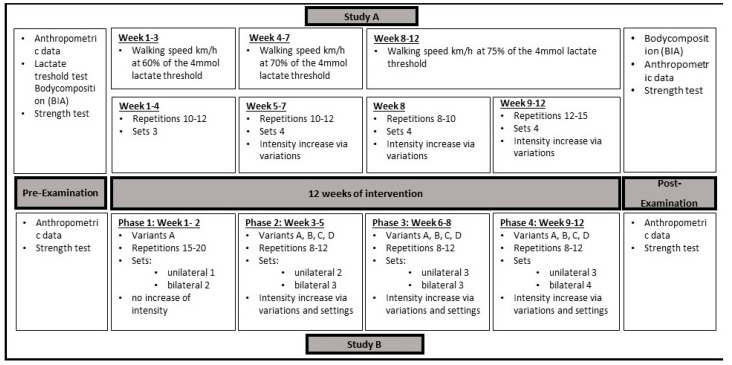
Study A and B protocols. Variants A–D describe the increase in difficulty by reducing the support base in each exercise variation.

Each woman received an individual parameter for endurance training. Over 3 weeks, for familiarization, walking training took place at a speed corresponding to 60% of the 4 mmol lactate threshold. Walking speed and heart rate were monitored by using sports watch Polar Ignite for training supervision. Subsequently, training was increased to 70% km/h of the 4 mmol lactate threshold for the following 4 weeks. Then, for the last 5 weeks, the training was at 75% km/h of the 4 mmol lactate threshold. All data were stored in the Polar Coach and tracked by the study management. Online strength training was offered twice a week via Cisco Webex Meetings (Cisco Systems GmbH). An alternate appointment was offered if participants were absent. The intervention and control groups completed the strength training together. All participants had to attend 80% of endurance training and 100% of strength training. Strength training consisted of bodyweight exercises such as squats, crunches, dips, and planks for all major muscle groups, such as M. quadriceps femoris, M. ischiocrurales, Mm. pectorales, M. triceps brachii, M. biceps brachii, Mm. glutei, and the trunk muscles. The gluteal and abdominal muscles must be constantly tensed to keep the body tense. The first 4 weeks were used for familiarization and a successive increase in intensity. Thus, we started with 10 repetitions in 3 sets, and increased to 12 repetitions in 3 sets. This was followed by an increase to 10 to 12 repetitions in 4 sets in the following 3 weeks. In the 8th week, a load–relief phase was scheduled with 8–10 repetitions in 4 sets before increasing to 12–15 repetitions in 4 sets in the last 4 weeks. In addition, the intensity of the exercises was increased through changes in execution. Each training session was organized as circuit training, so the strained muscle groups were changed and had time to relax. The cardiovascular system, however, was constantly strained. During the 12 weeks of the intervention, the women were not allowed to participate in other kind of sports (Figure 2).

#### 2.1.4. Nutritional Intervention

The intervention group received protein/carbohydrate supplementation consisting of 100 g of sour milk cheese (Käserei Loose) and 76 g of white bread immediately after each training session. The nutritional values of the meal were 36.1 g protein, 35.3 g carbohydrate, 3.5 g fat, and 321 kcal. Sour milk cheese was provided by Käserei Loose, Leppersdorf, Germany (Table 1).

### 2.2. Study B: Effect of Sling Training and Protein/Carbohydrate Supplementation in Elderly Men and Women

#### 2.2.1. Study Design and Participants

**Figure 3:**
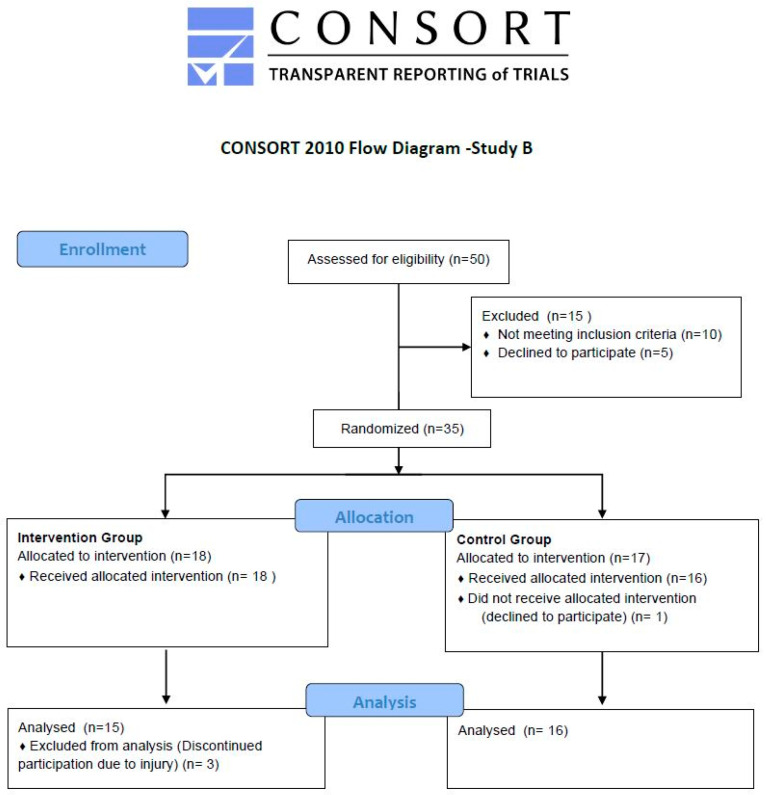
Consort flow diagram—Study B.

The study was designed as a randomized intervention study with two parallel groups (IG and CG). We included 35 participants in the randomization (Figure 3). Simple randomization was used, and care was taken to ensure an equal distribution ofparticipants. Stratification based on gender, age and weight data was performed during randomization. The sample size was determined on the basis of preliminary studies and the results of Gaedtke (2014). All participants completed sling training based on Gaedtke [35,36,37]. All men and women were recruited in the Ruhr area. Inclusion criteria were an age above 60 years and having been active in sports for at least one year. Exclusion criteria were an acute disease of the musculoskeletal system, cardiovascular diseases, and experience with sling training. After the study procedure had been communicated via telephone, and the inclusion and exclusion criteria had been checked, the interested participants received all information in paper form. Subsequently, an appointment was booked to sign the informed consent form, clarify existing questions, and start with the examinations. Prior to recruitment, the approval of the ethics committee of the German Sport University, Cologne (number 82/2015) was obtained. The study protocol was in accordance with the Declaration of Helsinki. The examinations and training sessions took place in a training center for seniors in Essen-Bochold and Gelsenkirchen-Mitte.

#### 2.2.2. Test Day

The maximal force for chest (chest press) and leg (leg press) strength was established via the repetition maximum according to Rühl [34,38]. The Swiss Olympic trunk test was also performed for the ventral, dorsal, and lateral trunk muscles. The following program was followed according to Tschopp [39,40].

After a 10 min warm-up, ventral, lateral, and dorsal trunk strength was tested. The participants always had a 10 min break between individual tests.

Ventral trunk strength: From a plank position, feet were lifted alternately while contact had to be maintained with a control bar on the head and glutes. The time for which the correct position could be maintained was measured.

Lateral trunk test: From lateral support, the pelvis was lowered and raised again, and contact with the control bar on the pelvis had to be repeatedly established. The seconds were counted.

Dorsal trunk test: From a prone position on a box, the upper body was lowered and raised again, and the control bars had to be touched. The seconds were counted.

#### 2.2.3. Training Intervention

The only training during the 12 weeks of intervention was sling training. Training took place three times per week (Monday, Wednesday, and Friday) and lasted 30 min. The whole group was divided into smaller groups of 3 to 6 to create a safe training situation. The intervention and control groups completed the training together. The training was divided into 4 training phases of 2 weeks. In the first phase of training, the participants were familiarized with the equipment, and the workout took place, so low intensity and high repetitions were used. Training control was performed with four different variations of an exercise (A–D), where A represented the easiest and D the most challenging variation. In the different variants, the difficulty was increased by reducing the support base (principle of the support base) [37].

Each training session included 7 exercises with 90 s rest between each exercise. The exercises were divided into:

- Two exercises for the upper body (rowing and chest press).
- Two exercises for the legs (squat and hip abduction).
- Two exercises for the trunk (crunches and side bend).
- One exercise for the entire ventral chain (body stretching).

The sequence of exercises was chosen so that one muscle group was not used twice in succession. Body tension is the basis of every exercise. The gluteal and abdominal muscles must be constantly tensed to keep the body in extension. In addition, the shoulders always remain low, and the neck relaxed. These points were emphasized in each unit.

The number of repetitions Increased after a subject had achieved two more repetitions on one exercise in the last set over two training sessions (progressive overload). This ensured progression in the training, which started with 8 repetitions and ended with 12. Once the 12 repetitions had been reached, the intensity was increased using the OMNI Res value. For this purpose, the trainer asked for a value between 1 and 10 after each set, where 1 meant very low effort and 10 meant very high effort [41]. On the basis of the training goal, 6 and 8 was the optimal intensity range. Using the settings, the suspensions or variant intensity could be increased if the value was less than 6 or decreased if the value was greater than 8 (Figure 2).

#### 2.2.4. Nutritional Intervention

After the training session, the intervention group received 100 g of sour milk cheese (Käserei Loose), 76 g of white bread, and 250 mL buttermilk immediately after each sling training session. The nutritional values of the meal were 44.6 g protein, 45.8 g carbohydrate, 5 g fat, and 416 kcal. Sour milk cheese and buttermilk were provided by Käserei Loose, Leppersdorf, Germany (Table 1). After each training session, all participants remained in the training center for 30 min to ensure that only the intervention group received the protein/carbohydrate supplementation.

### 2.3. Statistical Analysis

Strength data were normalized to body weight prior to further analysis. Subsequently, they were processed with principal component analysis (PCA). All variables had been mean centered and scaled to unity variance. The total variances of the datasets thus amounted to 4.0 (Study A) and 6.0 (Study B). PCA was performed on the data acquired before the intervention periods. Postintervention data then were submitted to PCA transformation using previously obtained coefficients. This procedure facilitated the detection of possible changes in the latent variables represented by the strength dataset.

The variables extracted with PCA were then analyzed with linear mixed-effect models (LME). As we were interested in the experimental effects of training intervention combined with supplementation, the fixed effects consistently encompassed these factors and their corresponding interaction term.

For Study B, the subjects were classified according to their adiposity status (BMI > 30). Adiposity served as an additional covariate with two levels (adipose: Adip+, not adipose: Adip−). Because participants in Study B were significantly more overweight and also obese compared to participants in Study A, we decided to analyze weight as a covariate. In addition, the interaction of adiposity and training intervention was included. The incorporation of higher-order interaction terms was not feasible due to the restricted sample size. Time (i.e., training intervention) grouped within individuals consistently served as a random effect. The used software was the latest version of statistical language R [42]. LMEs were fitted with the use of R’s standard nlme library [43]. The assessors were not blinded, but the data analysis staff were blinded in both studies.

## 3. Results

### 3.1. Study A

A total of 51 postmenopausal women (57.3 ± 3.0 years) finished the study (Table 2). Reasons for dropouts were diseases/injuries (N = 4), elevated hormone levels that did not meet the inclusion criteria (N = 4), and too much time expenditure (N = 4). None of the diseases or injuries were related to training.

Table 3 shows the changes in the strength and body composition of the intervention and control groups; all strength parameters could be increased. PCA showed that the individual strength parameters could be represented as a general strength score. There was a significant training effect, but the effect of the influence of supplementation was not significant. A change in body composition with a reduction in fat and an increase in muscle mass was also evident.

#### 3.1.1. PCA

The PCA yielded merely one significant component, i.e., one variable with variance larger than unity (PC1) that contained 0.65% of the total variance. Therefore, all four strength values (chest strength, leg strength, and left- and right-hand-grip strength) were combined into one strength value, the general strength score (GS).

#### 3.1.2. LME

**Figure 4:**
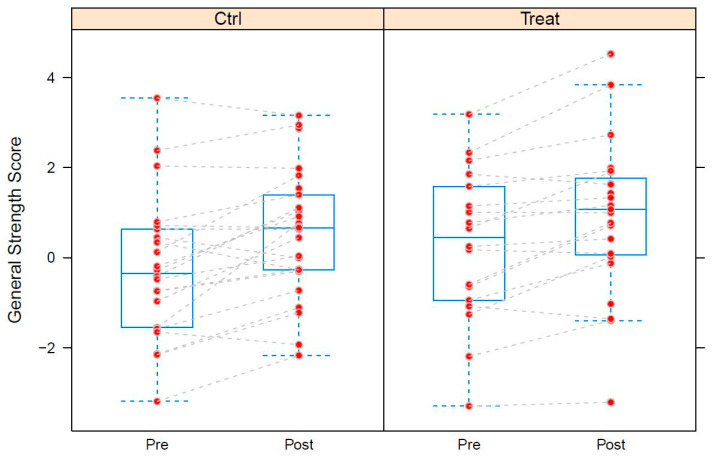
Box-plots of the General Strength Score (GS) calculated from the strength data of study A grouped by supplementation (Ctrl: control group; Treat: treatment group). Pre: measurement before training intervention; Post: measurements after training intervention. GS corresponds to the 1st principal component extracted from the strength data. See text for detailed explanation of the score. The box-plots indicate minima, 1st quartiles, medians, second quartiles and maxima, respectively. Maxima or minima falling beyond the median ± 1.5 times the respective interquartile range (“outliers“) are placed outside the whiskers. Dashed lines correspond to repeated measurements of the same subjects.

Figure 4 shows the changes in general Strength score (intervention group = Treat; control group = Ctrl). Table 4 shows the significant changes due to training. Training intervention had a positive and strongly significant effect o (+0.65, *p* ≤ 0.001) but supplementation had no detectable additional effect (ca. 0, *p* = ca. 0.85). Figure 4 shows significant increases in general strength.

### 3.2. Study B

A total of 31 participants comprising 6 men and 25 women finished the study (65.9 ± 4.9 years) (Table 5). We excluded 3 participants during the 12 weeks because of injuries. None of the diseases or injuries were related to training.

#### 3.2.1. PCA

Table 6 shows strength increase and body-weight decrease values. All participants gained strength and lost body weight. Using PCA, two strength scores (limb strength and trunk strength) could be formed from the six strength values (leg, chest, ventral-trunk, dorsal-trunk, and left and right lateral-trunk strength). PCA yielded two significant components, PC1 and PC2, with 0.55% and 0.25% of the total variance, respectively. Thus, the cumulative variance amounted to 80%.

#### 3.2.2. LME

**Figure 5:**
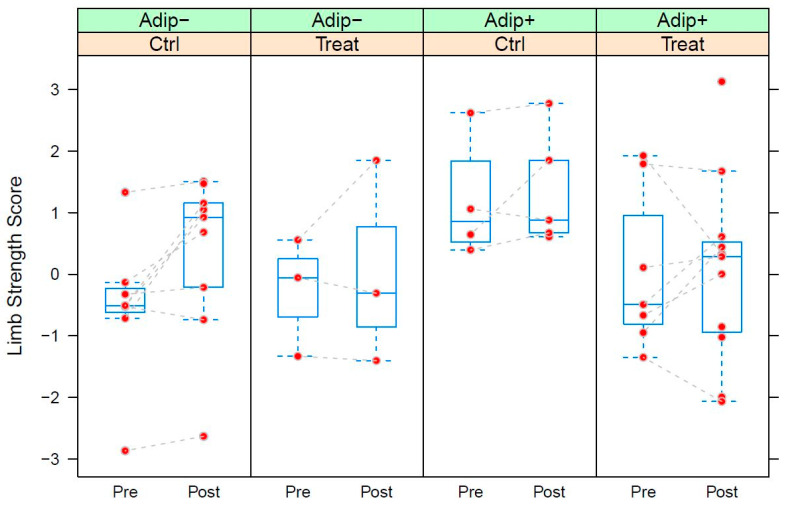
Box-plots of the Limb Strength Score (LS) calculated from the strength data of study B grouped by supplementation (Ctrl: control group; Treat: treatment group) within adiposity status (Adip−: BMI <30, Adip+: BMI >= 30). Pre: measurement before training intervention; Post: measurements after training intervention. LS corresponds to the 2nd principal component extracted from the strength data. See text for detailed explanation of the score. See capation of Figure 4 for details of the box-plots.

**Figure 6:**
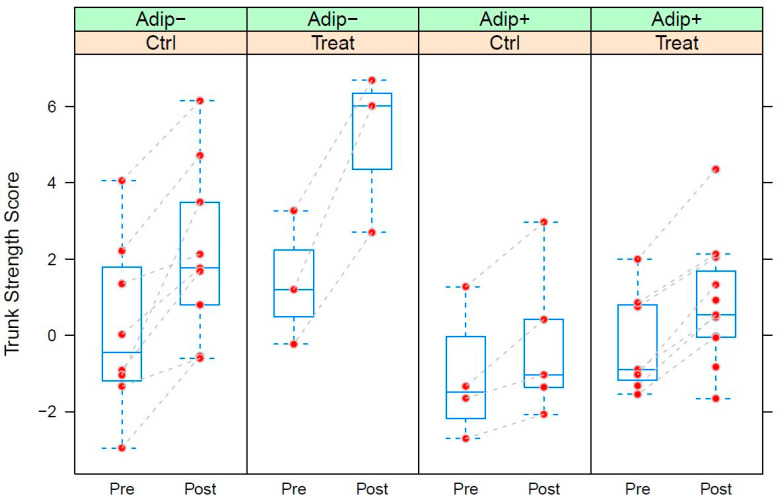
Box-plots of the Trunk Strength Score (TS) calculated from the strength data of study B grouped by supplementation (Ctrl: control group; Treat: treatment group) within adiposity status (Adip−: BMI < 30, Adip+: BMI >= 30). Pre: measurement before training intervention; Post: measurements after training intervention. TS corresponds to the 1st principal component extracted from the strength data. See text for detailed explanation of the score. See caption of Figure 4 for details of the box-plots.

Table 7 and Table 8 show the parameters of the LMEs fitted to trunk-strength (TS) and limb-strength (LS) data, respectively. TS significantly increased during the training intervention (+2.305, *p* ≤ 0.001). While adipose subjects exhibited lower starting values (−1.683, *p* ≤ 0.05), they also react significantly more weakly to the training intervention (−1.513, *p* ≤ 0.01). Dietary supplementation yielded an additional and significant positive effect on TS (+0.950, *p* ≤ 0.05) (Table 7 and Figure 5). LS significantly increased after the training intervention (+0.753, *p* < 0.05). Apart from a weak initial trend of adipose subjects showing stronger values (+0.942, *p* = 0.096), there were no further significant terms in the model (Table 8 and Figure 6).

## 4. Discussion

We compared two different training types in combination with protein/carbohydrate supplementation via food immediately after training in postmenopausal women, and elderly men and women. Both studies showed a positive effect of training; sling training increased limb and especially trunk strength in elderly men and women. Gaedtke showed significant results on chest-muscle strength in elderly people through sling training [35]. Various studies showed a positive effect of sling training on muscle mass in general and the trunk muscles in particular [36,44,45,46,47]. Trunk-muscle strength was more promoted in the IG after the consumption of sour milk cheese, bread, and buttermilk than that in the CG. We were not able to show this effect in limb strength. However, training was significantly more effective in the intervention group. Sling training is common as a treatment for lower-back pain, which is often triggered by a deficit in the trunk muscles. Local trunk muscles are a possible explanation for the larger effects in trunk strength [48,49]. Protein/carbohydrate supplementation seemed to be able to intensify these positive effects (Figure 5), perhaps due to the proregenerative effects that Isenmann and colleagues described [27]. The elderly participants regularly use their leg and chest muscles in everyday life, so the proregenerative effects could not show a large effect. However, as aging trunk muscles slim down, sling training with a focus on trunk stabilization, like our training, is a great challenge and requires these muscles. The training was offered three times per week. Therefore, there may be proregenerative effects, such as an increase in insulin serum concentration, a decrease in proinflammatory markers, and an increase in anti-inflammatory markers. Isenmann and colleagues showed these effects in young men after a similar protein/carbohydrate supplementation via food immediately after training [27]. The positive proregenerative effect of protein/carbohydrate supplementation after training was also shown by other authors [26,50]. Zawadzki and colleagues showed that the administration of a protein/carbohydrate combination right after training could enhance glycogen storage in muscles, which contributes to faster recovery. Leg and chest strength also increased over the 3 months, but this effect was not increased via protein/carbohydrate intake [50].

The significant training effect was less pronounced in participants with a higher BMI (>30) than that in participants with a lower BMI (Figure 5 and Figure 6). A possible explanation could be that, with greater body weight, the sling exercises could not be performed as well or could only in simpler variations, which could have caused a reduced training effect. Morat et al. showed that variations in body angle while training intensify the sling training [47]. On the other hand, the participant group was more likely to have the high body fat percentage that caused the high BMI. Since untrained and overweight individuals usually experience faster and greater effects with the same training than those of persons with normal weight, such effects were also more likely here [51]. Therefore, it is reasonable to assume that sling training was not as effective for heavier participants. In summary, a significant increase in strength was achieved in the seniors as a result of the training, which is a very positive effect, especially with regard to the risk of sarcopenia. Supplementation with food in the form of an evening meal increased trunk strength more significantly, which is an important aspect with regard to frailty in old age.

The combination of endurance and strength training for postmenopausal women could increase strength and made the women feel more fit. We showed effects in whole body strength, which consists of chest, leg, and hand-grip strength. The effect on the hand grip is important for postmenopausal women because many of them have low bone and low muscle mass, which comes with a low hand grip. In addition, low hand-grip strength is associated with a low quality of life [52]. Using principal component analysis, we and other authors showed that hand-grip strength could be used as an indicator of overall body strength [53]. So, these findings are in line with the increase in and the role of hand-grip strength. Thus, the increase in hand-grip strength may explain the increase in the subjective fitness status of the participants. Leg and chest strength was analyzed via the repetition maximum, which is a possible reason for the lesser effects. In this repetition method, the one repetition maximum (1 RM) is not tested, but calculated on the basis of weight and repetitions. This method can be used especially for untrained and inexperienced people, and in medical training therapy [54]. In a heterogeneous group (experienced and inexperienced or trained and untrained participants), however, this can lead to errors or unequal results. We were not able to demonstrate a significant influence of supplementation even if hand strength increased more significantly.

In Study B, there was a significant decrease in body weight; in Study A, we were unable to show this. However, participants in Study A with a BMI of 25.1 were normal to slightly overweight compared to the participants in Study B (BMI 30.9). In addition, in Study B, strength training was performed 3 times per week, whereas in Study A, moderate and fat-metabolism-oriented endurance training was performed twice per week, and strength training only once per week. Thus, endurance training had an effective effect on fat mass, whereas strength training had a faster effect on body weight [5]. Here, the exact determination of body composition in Study B would have been helpful.

The limitations of our study are the missing analysis of insulin, skeletal-muscle creatine kinase, myoglobin, and serum cytokine levels in both studies that would have contributed to proving the proregenerative effect. Nutritional and protein statuses were not recorded before and during training in either study, which would have been useful in identifying possible deficiencies in the supply or oversupply of dietary protein, especially since the effect of protein/carbohydrate supplementation was so pronounced in older participants. One hypothesis here is that the postmenopausal women consume sufficient protein in their diet, while the seniors showed a deficit. In Study B, protein/carbohydrate supplementation was provided as a common meal in the evening after training: sour milk cheese, bread, and buttermilk. In Study A, this effect was absent because no communal meal was possible after training due to the COVID-19 pandemic. Thus, some participants trained in the morning and supplemented the protein/carbohydrate combination afterwards, and others trained in the evening. Due to the different training times, the supplementation was not taken together as a meal, which may have led to participants taking the supplementation in addition, while others replaced a meal with it. This could explain the missing change in body weight. The supervision of the consumption of sour milk cheese and bread after each training session was also lacking, which presumably reduced compliance among the women. In Study B, body composition was not determined, so conclusions could be drawn about the changes in body weight, but not about the exact body composition. These parameters would have been useful to see the influence of training and protein/carbohydrate intake on muscle and fat mass. Since the body weight of the subjects in Study B were significantly higher (85.4 ± 15.6 kg) than those in Study A (69.7 ± 12.7 kg), the exact body composition would have been an interesting parameter to compare baseline muscle and fat mass because a significantly lower baseline value would be assumed for the seniors, according to [17,55]. Study B also showed that the training effect was less pronounced in participants with a higher BMI (>30) than that in lighter participants. This may have been due to the nature of the training, as the participants’ entire weight had to be carried on the slings. This is much more difficult with a greater body weight and may mean, for example, that the exercises could only be increased more slowly.

## 5. Conclusions

Our results indicated that a combination of training and protein/carbohydrate supplementation via food directly after training may be a suitable strategy to counteract the age-related loss of trunk strength in seniors. The combination of strength and endurance training in postmenopausal women, and sling training in older subjects led to improved strength. We only demonstrated the influence of protein/carbohydrate supplementation regarding specific parameters, but this may have been due to methodological limitations and the COVID-19 pandemic. In the future, body composition should be taken into account, and the meal character of protein/carbohydrate supplementation should be adhered to. In addition, it would be interesting to perform Study A with a group of participants with a BMI above 30.

## Figures and Tables

**Table 1:** Nutritional values in Studies A and B.

**Study A**	**100 g Sour Milk Cheese and 76 g White Bread**
Protein (g)	36.1
carbohydrate (g)	35.3
fat (g)	3.5
kcal	321
**Study B**	**100 g Sour Milk Cheese and 76 g White Bread and 250 mL Buttermilk**
Protein (g)	44.6
carbohydrate (g)	45.8
fat (g)	5
kcal	416

**Table 2:** Anthropometric data with mean and standard deviation—Study A.

	Total Sample	Intervention Group	Control Group
Study A	N = 51	N = 24	N = 28
Age (years)	57.3 ± 3.0	57.9 ± 3.3	56.8 ± 2.8
Height (cm)	167 ± 7.3	169.8 ± 6.9	164.6 ± 6.7
Weight (kg)	69.7 ± 12.7	70.8 ± 15.2	68.8 ± 10.3
BMI	25.1 ± 4.4	24.5 ± 4.7	25.5 ± 4.2

**Table 3:** Strength parameters and body composition showing the mean and standard deviation—Study A.

N = 51	Intervention Group		Control Group	
	Pre Intervention	Post Intervention	Pre Intervention	Post Intervention
Strength				
Leg (kg)	89.9 ± 20.9	95.9 ± 24.2	91.4 ± 26.4	105.0 ± 25.9
Chest (kg)	28.0 ± 8.2	31.9 ± 8.4	25.5 ± 5.6	27.7 ± 5.7
Hand-grip right (kg)	28.9 ± 4.7	31.3 ± 4.0	27.8 ± 4.2	29.0 ± 3.9
Hand-grip left (kg)	27.9 ± 4.5	29.1 ± 3.9	26.8 ± 4.5	27.5 ± 4.3
Hand-grip sum (kg)	51.1 ± 18.9	54.2 ± 18.9	53.5 ± 9.4	56.5 ± 7.6
Body composition				
Body weight	70.8 ± 15.2	70.5 ± 15.5	68.8 ± 10.5	68.1 ± 10.1
Muscle mass	19.1 ± 2.4	19.3 ± 2.5	18.8 ± 1.8	19.2 ± 2.0
Fat mass	25.6 ± 11.0	25.2 ± 11.1	24.4 ± 7.7	23.4 ± 7.1

**Table 4:** LME parameters fitted to GS data.

	Value	Std. Error	DF	t-Value	*p*-Value
(Intercept)	−0.0888	0.321	46	−0.276	0.784
TimePost	0.649	0.15	38	4.32	0.000108
GrpTreat	0.398	0.467	46	0.851	0.399
TreatPost × GrpTreat	−0.0427	0.224	38	−0.191	0.85

**Table 5:** Anthropometric data showing mean and standard deviation—Study B.

	Total Sample	Intervention Group	Control Group
Study B	N = 31	N = 15	N = 16
Age (years)	65.9 ± 4.9	67.7 ± 5.9	64.2 ± 3.2
Height (cm)	166.1 ± 8.5	165.3 ± 9.2	166.9 ± 7.9
Weight (kg)	85.4 ± 15.6	87.3 ± 14.9	83.6 ± 15.5
BMI	30.9 ± 5.1	31.8 ± 4.1	30.0 ± 5.7

**Table 6:** Changes in the strength and body weight of control and intervention groups with the mean and standard deviation.

N = 31	Intervention Group		Control Group	
	Preintervention	Postintervention	Preintervention	Postintervention
Leg strength (kg)	117.0 ± 41.7	142.9 ± 50.2	105.3 ± 32.2	130.8 ± 32.3
Chest strength (kg)	39.7 ± 14.8	46.5 ± 15.9	30.4 ± 26.2	44.9 ± 30.8
Ventral-trunk strength (sec)	30.3 ± 18.1	49.4 ± 21.1	27.8 ± 4.2	29.0 ± 3.9
Lateral-trunk strength—right	12.9 ± 11.7	30.6 ± 16.2	12.4 ± 11.4	19.8 ± 13.9
Lateral-trunk strength—left	19.4 ± 13.5	30.7 ± 13.0	13.1 ± 9.3	21.8 ± 12.4
Dorsal-trunk strength	65.2 ± 41.3	79.6 ± 49.8	62.5 ± 26.4	85.6 ± 31.7
Body weight	87.2 ± 14.9	85.7 ± 14.3	83.6 ± 15.4	82.6 ± 15.2

**Table 7:** LME parameters fitted to the trunk strength score (PC1, TS) of Study B.

	Value	Std. Error	DF	t-Value	*p*-Value
(Intercept)	0.157	0.551	25	0.285	0.778
TimePost	2.305	0.291	19	7.921	0.000
AdipAdip+	−1.683	0.763	25	−2.207	0.037
GrpTreat	0.856	0.758	25	1.130	0.269
TimePost × AdipAdip+	−1.513	0.407	19	−3.718	0.001
TimePost × GrpTreat	0.950	0.407	19	2.331	0.031

**Table 8:** LME parameters fitted to the limb-strength score (PC2, LS) of Study B.

	Value	Std. Error	DF	t-Value	*p*-Value
(Intercept)	−0.247	0.393	25	−0.628	0.536
TimePost	0.753	0.274	19	2.751	0.013
AdipAdip+	0.942	0.545	25	1.728	0.096
GrpTreat	−0.562	0.543	25	−1.034	0.311
TimePost × AdipAdip+	−0.364	0.383	19	−0.952	0.353
TimePost × GrpTreat	−0.352	0.383	19	−0.917	0.371

## Data Availability

The data presented in this study are available on request from the corresponding author.
